# The Efficacy of Ingesting Water on Thermoregulatory Responses and Running Performance in a Warm-Humid Condition

**DOI:** 10.3389/fphys.2019.00507

**Published:** 2019-05-07

**Authors:** Ahmad Munir Che Muhamed, Hazwani Ahmad Yusof, Stephen R. Stannard, Toby Mündel, Martin William Thompson

**Affiliations:** ^1^Lifestyle Science Cluster, Advanced Medical and Dental Institute, Universiti Sains Malaysia, Penang, Malaysia; ^2^School of Sport, Exercise and Nutrition, Massey University, Palmerston North, New Zealand; ^3^Discipline of Exercise and Sport Science, Faculty of Health Science, The University of Sydney, Sydney, NSW, Australia

**Keywords:** fluid ingestion, thermoregulation, circulation, relative humidity, running exercise

## Abstract

The understanding that fluid ingestion attenuates thermoregulatory and circulatory stress during exercise in the heat was based on studies conducted in relatively dry (∼50% RH) environments. It remains undetermined whether similar effects occur during exercise in a warm and more humid environment, where evaporative capacity is reduced. Nine well-trained, unacclimatised male runners were randomly assigned to perform four experimental trials where they ran for 60 min at an intensity of 70% VO_2_max followed by an incremental exercise test until volitional exhaustion. The four trials consisted of non-fluid ingestion (NF) and fluid ingestion (FI) in a warm-dry (WD) and warm-humid condition (WH). Time to exhaustion (TTE), body temperature (T_b_), whole body sweat rate, partitional calorimetry measures, heart rate and plasma volume were recorded during exercise. There was no significant difference in T_b_ following 60 min of exercise in FI and NF trial within both WD (37.3°C ± 0.4 vs. 37.4°C ± 0.3; *p* > 0.05) and WH conditions (38.0°C ± 0.4 vs. 38.1°C ± 0.4; *p* > 0.05). The TTE was similar between FI and NF trials in both WH and WD, whereas exercise capacity was significantly shorter in WH than WD (9.1 ± 2.8 min vs. 12.7 ± 2.4 min, respectively; *p* = 0.01). Fluid ingestion failed to provide any ergogenic benefit in attenuating thermoregulatory and circulatory stress during exercise in the WH and WD conditions. Consequently, exercise performance was not enhanced with fluid ingestion in the warm-humid condition, although the humid environment detrimentally affected exercise endurance.

## Introduction

Recent evidence demonstrates a reduced physical capacity of trained individuals during exercise in the heat as relative humidity (RH) increases (Maughan et al., 2012; Moyen et al., 2014; Che Muhamed et al., 2016). This observation is attributed to a greater thermoregulatory and circulatory strain (Moyen et al., 2014; Che Muhamed et al., 2016), as the evaporative capacity of the environment (E_max_) reduces, leading to a decline in sweating efficiency and increased area of skin wettedness and the subsequent rise in core temperature (Candas et al., 1979; Frye and Kamon, 1983; Alber-Wallerström and Holmér, 1985).

Numerous recommendations encourage athletes to ingest sufficient fluid in order to prevent a loss of >2% of their body weight during exercise (Sawka et al., 2007, 2015). This recommendation was based on laboratory studies demonstrating that a body mass loss of more than 2% impairs aerobic exercise performance in a thermally stressful environment (Montain and Coyle, 1992; Sawka et al., 2015). On the other hand, numerous publications have refuted the claim that aerobic exercise performance is degraded with increased (>2%) dehydration (Noakes, 2007; Goulet, 2011, 2013; Wall et al., 2013). A meta-analysis by Goulet (2011) had highlighted that exercise-induced dehydration by up to 4% of body weight loss does not alter exercise performance. Instead, drinking according to the dictate of thirst will potentially improve exercise performance (Noakes, 2007). While the debate on the efficacy of fluid ingestion in minimizing the decline of aerobic exercise performance in ambient heat continues, the value of fluid ingestion during humid conditions is not well understood.

Early reports of an attenuation in hyperthermia with fluid ingestion are based on studies conducted in a hot and relatively dry (∼50% RH) environment with the large E_max_ allowing for efficient sweat evaporation (Montain and Coyle, 1992; Below et al., 1995). By contrast, in a humid environment with a lower E_max_, sweat will drip off the skin instead of evaporating (Candas et al., 1979; Frye and Kamon, 1983; Alber-Wallerström and Holmér, 1985) and so “ineffective” fluid loss through sweating is much greater. Therefore, the proportion that fluid ingestion contributes to thermoregulation via increasing sweating rate would also be less. These observations on the sweating response and sweating efficiency might question the efficacy of fluid ingestion during prolonged exercise in a warm-humid condition. To date, Kay and Marino (2003) have reported that self-paced exercise performance in a hot-humid environment was not improved with fluid ingestion. In addition, Lee et al. (2010), had reported that fluid ingestion did not alter thermoregulatory responses during field-based experiments among military personnel. Therefore, the purpose of this study was to investigate the efficacy of ingesting plain water in attenuating the thermoregulatory and circulatory stress during prolonged moderate-high intensity exercise under a warm-dry and warm-humid environment and its consequences on running performance.

## Materials and Methods

### Participants

Nine well-trained, unacclimatised male runners who regularly participated in middle and long distance running events volunteered for this study. The subjects’ characteristics were (mean ± SD): age, 32 ± 4 years; height, 183 ± 6 cm; weight, 72 ± 4 kg; body surface area, 1.9 ± 0.1 m^2^; percent body fat, 11 ± 6%; VO_2_max, 62 ± 5 ml kg^-1^ min^-1^. Participants were briefed on the experimental protocol and testing procedures before providing written informed consent to participate. The University of Sydney Human Ethics Committee approved the experimental protocol for this study (Ref. No: 99/05/46), which conformed to the current Declaration of Helsinki guidelines. All experiments were conducted in a purpose-built environmental chamber during the autumn and winter season in Sydney, Australia. The average outdoor temperature during the experimental trials was 20 to 22°C in autumn and 7 to 10°C during winter. None of the participants were accustomed to exercise in the conditions simulated in this study.

#### Preliminary Testing and Familiarization

Each participant visited the environmental chamber and was familiarized with the exercise protocol, equipment, and measurement procedures used in the study. This was followed by a preliminary testing session during which anthropometric measures, including body height, weight, composition, and surface area were determined. Body surface area was calculated using the method of DuBois and DuBois (1916). Percent body fat was measured using the hydrodensitometry underwater weighing technique described by Siri (1961).

Each participant then performed a running economy test followed by a maximal exercise test. The running economy test required each subject to run at four submaximal velocities of 10, 12, 14, and 16 km h^-1^, respectively, for 4 min per stage. On completion of the submaximal running, participants engaged in an active recovery, where they walked for 5 min at a speed of 5 km h^-1^. This was followed by a graded exercise test to determine maximal oxygen uptake (VO_2_max), where the participant ran at a fixed speed of 12 km h^-1^ with the treadmill gradient elevated by 2% every 2 min until volitional fatigue was attained. Oxygen consumption and heart rate were taken throughout the exercise duration. Post-exercise, a linear regression line was plotted between submaximal steady state oxygen consumption and treadmill velocity to determine the participant’s running speed that elicited an intensity of 70% VO_2_max, which was used for the individual’s experimental trials for each subsequent testing session. The preliminary and familiarization sessions were conducted in a thermoneutral environment (20°C, 40% RH).

Prior to reporting to the laboratory for testing, each participant was reminded to refrain from heavy exercise and alcohol consumption the day before testing and to avoid caffeine consumption for 12 h before the test. They were also told to maintain a similar training routine throughout the duration of the study. Participants were asked to keep a 24 h food diary before testing and replicate their diet before subsequent visits to the laboratory. To ensure that participants were euhydrated at the onset of exercise, they were asked to ingest 6 ml of water per kg lean body mass at 2 h intervals (excluding when asleep) during the day before the test as well as the morning of testing.

### Experimental Protocol

Each participant ran for 60 min at a speed eliciting an intensity of 70% VO_2_max. Immediately thereafter, participants continued running at the same velocity while the treadmill gradient was elevated by 2% every 2 min until volitional exhaustion, defined as the point at which participants could no longer maintain the pace of the treadmill, upon which the test was terminated. During the 60 min submaximal exercise, the treadmill was briefly stopped (1 min) at 30 and 60 min to allow time for the determination of the participant’s body mass. The air speed set for each trial was matched to the individual running speed and thus simulated the effect of air resistance on a calm day outdoors. Each test session was separated by a week apart to minimize acclimation effects as well as to provide adequate recovery.

The experiment started with participants being randomly assigned to perform the running exercise without ingesting water (non-fluid ingestion trial; NF) across two environmental conditions of warm-dry (WD: 30°C and 24% RH) and warm-humid (WH: 30°C and 71% RH). Following the NF trial, participants were randomly assigned to the same exercise regimen while being allowed to ingest water (fluid ingestion trial; FI) across the WD and WH conditions. The NF trials enabled the determination of each individual subject’s sweat rate within the specific environmental condition for the calculation of the fluid volume needed to replace 80% of sweat loss during the FI trial.

At least 1 week elapsed between each of the subject’s four scheduled visits to the laboratory, in order to minimize any residual effects from the previous visit. To avoid any psychological apprehension, subjects were not informed of the environmental condition on the day of the test.

#### Fluid Ingestion Protocol

In the FI trial, water amounting to 80% of total sweat loss from the NF trial was equally divided into five aliquots and provided to participants during submaximal exercise, starting at 10 min and followed at every 10 min interval with the final ingestion at 50 min. The volume of fluid ingestion in this study which was based on 80% of total sweat loss was about 1 L (ranged between 1.12 and 0.96 L). This amount of fluid ingestion was consistent with the water requirements for an hour of running exercise at a similar metabolic rate in a hot and humid condition as previously described by Sawka and Pandolf (1990). The temperature of the water ingested at each time interval was maintained at 15°C.

### Measurements

As an index of core temperature, rectal temperature (T_re_) was measured by a thermistor probe (YSI 400 series; Mallinckrodt Medical, St. Louis, MO, United States) inserted 12 cm beyond the anal sphincter and data were recorded on a portable data logger (T-logger; The University of Sydney, Sydney, NSW, Australia). Skin temperature was measured at four different sites (left shoulder, left chest, right mid-thigh, and right mid-shin) using thermistor probes (YSI 409 Series). Rectal and skin temperatures were sampled at 1 min intervals. Weighted mean skin temperature (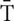
_sk_) was calculated from the four sites (Ramanathan, 1964). Mean body temperature (T_b_) was calculated using the equation developed by Colin et al. (1971) as T_b_ = 0.79 (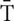
_re_) + 0.21 (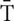
_sk_). Both rectal and skin thermistor probes used in the study were calibrated before and after the study in a water bath with temperature ranging from 15 to 50°C; calculated accuracies were ± 0.05 and ±0.01, respectively.

Expired respiratory gas was obtained using the Douglas bag method at rest and at every 10 min intervals of the submaximal exercise and analyzed for fractions of O_2_ and CO_2_ concentration using gas sensors and analyzers (O_2_ analyzer, Ametek S-3A/I and CO_2_ analyzers, Ametek CD-3A, Applied Electrochemistry Ametek Inc., Thermox Instruments Division). The gas analyzers were calibrated with known calibration gasses prior to each testing session.

Cardiac output during the steady state exercise phase was determined at 10, 30 and 60 min by the CO_2_ rebreathing method (Collier, 1956). Stroke volume was then calculated using the Fick equation. Heart rate was continuously monitored telemetrically via a Polar transmitter-receiver (Polar Vantage XL, Polar Electro, Kempele, Finland) and recorded at rest, during submaximal exercise at 5 min intervals and at volitional exhaustion.

Whole body sweating rate (WBSR) during steady state exercise was calculated as the difference in pre and post 60 min exercise nude body weight with correction for respiratory moisture loss (Mitchell et al., 1972) and incorporating volume of fluid consumed. Participant’s perception of the difficulty of the exercise effort was recorded based on the 6–20 point RPE scale (Borg, 1982) and was recorded at 10 min intervals.

A partitional calorimetry software program developed by Atkins and Thompson (2000) was used to estimate the magnitude and avenue of body heat gain and heat loss during each of the prolonged exercise bouts. Partitional calorimetry calculations were based on measures taken within the last 30 min of submaximal exercise of each trial. Evaporative heat loss in W m^-2^ was estimated from the classic body heat balance equation of E = M – W (Eres + Cres) – R – C – S, where M is metabolic heat production, W is external work, Eres + Cres is heat transfer via the respiratory tract, R is radiative heat loss, C is convective heat loss, and S is body heat storage. S was estimated using the following equation: [3474 × wt × (T_b_ final – T_b_ initial) × t^-1^] × A_D_^-1^ (W m^-2^), where 3474 is the average specific heat of body tissue (J kg^-1^ °C^-1^), wt is body mass (kg), T_b_ is mean body temperature (°C), t is exercise time (sec), and A_D_ is body surface area (m^2^). Estimation of tissue heat conductance (K) was derived from the method of Davies (1979) as K = H_sk_/[(T_re_-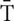
_sk_)^∗^A_D_]^-1^(Wm^-2°^C^-1^), where H_sk_ is the heat dissipated from the skin (E + R + C), Tre is rectal temperature, T_sk_ is mean skin temperature, and A_D_ is the DuBois body surface area in m^-2^. Convective and radiative heat loss in W m^-2^ were estimated using the equations found in Fanger (1970) and McIntyre (1980), respectively.

### Blood Sampling and Analysis

Venous blood was sampled at rest and 10, 30, and 60 min of submaximal exercise and were immediately stored for analysis of hematocrit and hemoglobin (CIBA-Corning 800 Series). Analysis for hematocrit percentage and hemoglobin concentration were analyzed in triplicate and used to estimate percentage changes in resting plasma volume based on the method of Dill and Costill (1974).

### Statistical Analysis

A two way (humidity x fluid treatment) repeated measures analysis of variance (ANOVA) was used to compare the differences between the means of the data measured in this study. If a primary significant difference was observed, the *post hoc* Tukey’s paired *t*-test with a Bonferroni correction for multiple comparisons was used to detect where the differences occurred. A Huynh-Feldt correction was applied to adjust the degrees of freedom when the test of sphericity was significant. Statistics were analyzed using SPSS statistical software (V22.0, Chicago, IL, United States), with statistical significance accepted at an ∞ level of 0.05. Data reported are presented as means ± SD.

## Results

### Steady-State Exercise

**FIGURE 1:**
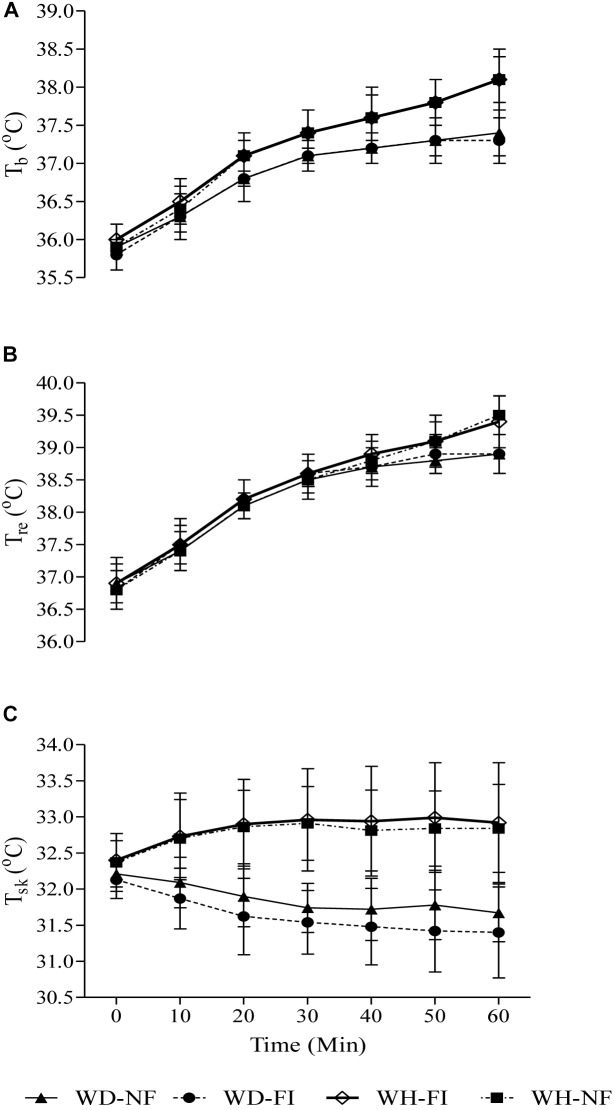
Mean body temperature **(A)**, rectal temperature **(B)**, and weighted mean skin temperature **(C)** responses during the steady state exercise in the fluid ingestion (FI) and non-fluid ingestion trial (NF) in a warm-dry (WD) and warm-humid (WH) condition.

Responses of T_re_, T_sk_ and T_b_ can be seen in Figure 1. ANOVA revealed no significant main effect of fluid ingestion on T_re_, T_sk_ or T_b_ (all *p* > 0.2), however, a main effect of humidity was observed for all (all *p* < 0.02) during the steady state exercise. At the end of steady state exercise, a significantly higher T_b_ was recorded in WH as compared with WD for both FI (38.0°C ± 0.4 vs. 37.3°C ± 0.3; *p* < 0.01) and NF trials (38.1°C ± 0.4 vs. 37.4°C ± 0.3; *p* < 0.01).

WBSR differed between fluid trials as a function of ambient humidity (fluid ingestion x ambient humidity: *p* < 0.01), such that NF in WD resulted in similar WBSR during exercise when compared with FI (24.5 g min^-1^ ± 2.3 vs. 24.3 g min^-1^ ± 2.3, respectively; *p* = 0.6), whilst in WH WBSR was significantly higher in FI compared to NF (26.1 g min^-1^ ± 4.8 vs. 22.7 g min^-1^ ± 3.4, respectively; *p* = 0.01). Percent body weight deficit was significantly larger in the NF as compared with the FI trial (*p* < 0.01), within both WD (2.0 ± 0.2 vs. 0.4 ± 0.1, respectively) and WH environments (1.8 ± 0.3 vs. 0.6 ± 0.2), although ambient humidity had no effect (*p* = 0.6). The change in PV (measured between 10th and 60th minutes) was similar between FI and NF trials (2.1 ± 1.8% vs. 4.2 ± 3.0%, respectively; *p* = 0.1) although the change in PV differed as a consequence of ambient humidity (2.0 ± 1.4% vs. 4.2 ± 2.9%, respectively; *p* = 0.04).

There was no significant effect of fluid ingestion on RPE during exercise (*p* = 0.4), however, ambient humidity significantly affected RPE (*p* < 0.01). Participants reported exercise as being harder to perform in the WH environment as compared with WD, respectively, for both NF (16 ± 2 vs. 14 ± 2, respectively) and FI trials (15 ± 2 vs. 13 ± 2, respectively).

ANOVA revealed a significantly higher S during NF than FI (6 ± 7 W m^-2^; *p* = 0.03) and WH than WD (22 ± 9 W m^-2^; *p* < 0.01). M was significantly higher during WH than WD (23 ± 27 W m^-2^; *p* = 0.03) with no effect of fluid ingestion (*p* = 0.3), whilst E was significantly higher during FI than NF (14 ± 15 W m^-2^; *p* = 0.03) with no effect of ambient humidity (*p* = 0.2). K and C + R were both higher during WH than WD (22 ± 12 and 25 ± 8 W m^-2^, respectively; both *p* < 0.01) with no effect of fluid ingestion (both *p* > 0.2).

**FIGURE 2:**
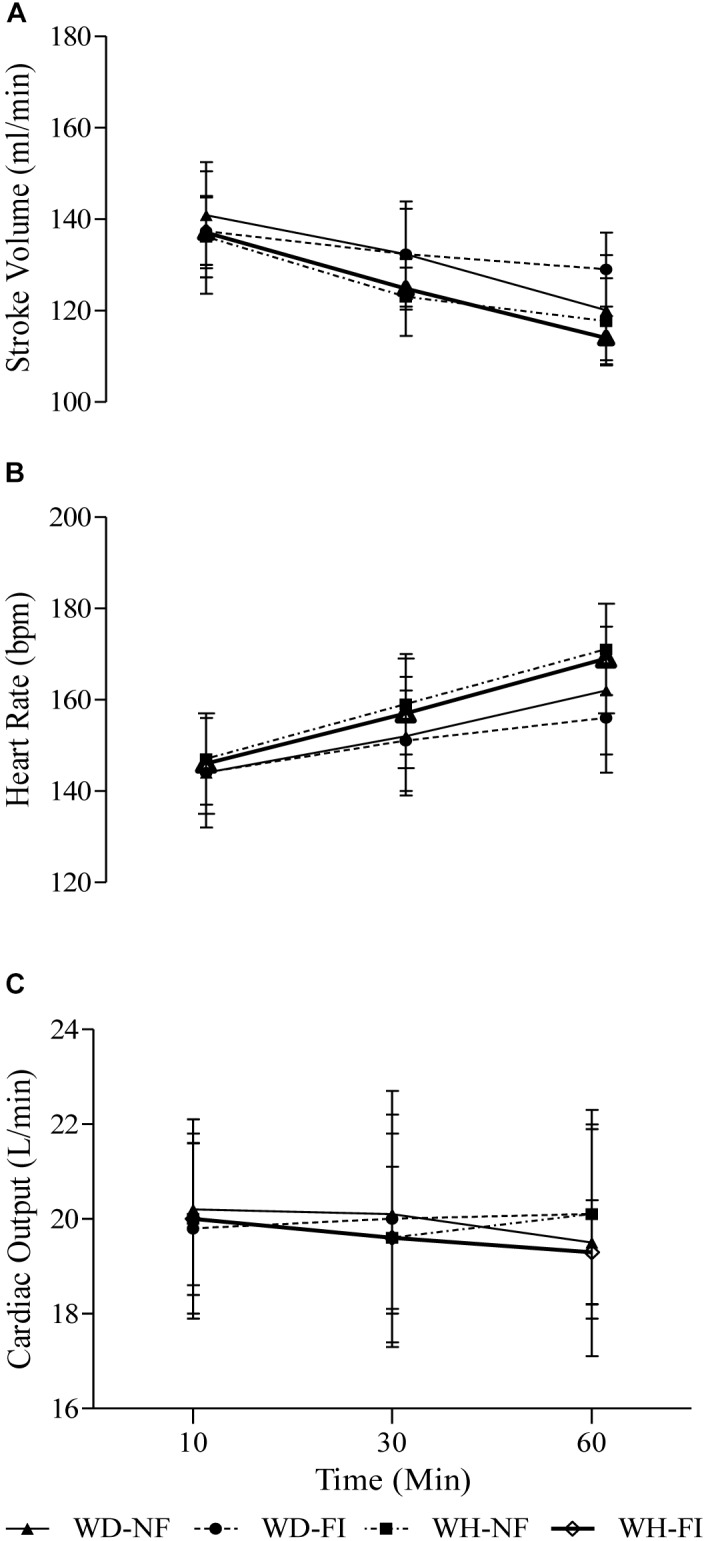
Stroke volume **(A)**, heart rate **(B)**, and cardiac output **(C)** during the steady state exercise in the fluid ingestion (FI) and non-fluid ingestion trial (NF) in a warm-dry (WD) and warm-humid (WH) condition.

The circulatory responses can be seen in Figure 2. ANOVA revealed a significant effect of fluid ingested (*p* = 0.01) and ambient humidity (*p* < 0.01) on heart rate, such that on average FI reduced HR by 2 ± 2 beats min^-1^ whilst on average the WH environment increased HR by 7 ± 5 beats min^-1^. Stroke volume was lower during WH than WD by 7 ± 5 ml (*p* < 0.01) with no effect of fluid ingested (*p* = 0.7). No effects of fluid ingestion or ambient humidity were observed on cardiac output(both *p* > 0.5).

### Graded Exercise to Exhaustion

**FIGURE 3:**
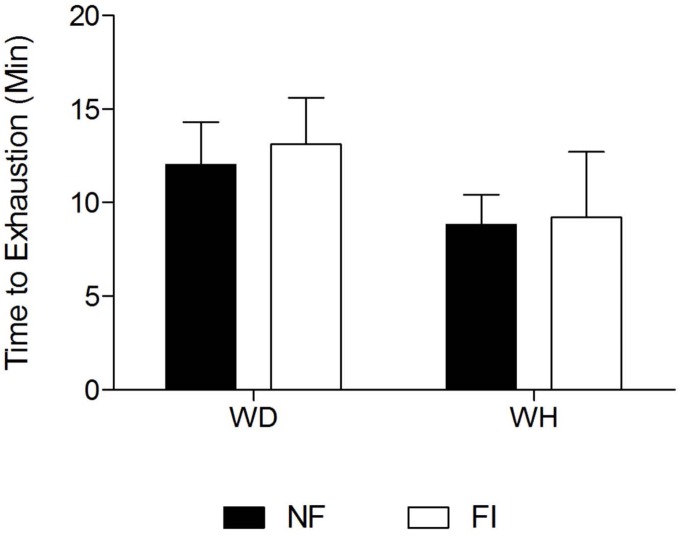
Mean Time to Exhaustion (TTE) during the graded exercise phase in the fluid ingestion (FI) and non-fluid ingestion trial (NF) in a warm-dry (WD) and warm-humid (WH) condition.

Fluid ingestion did not have any effect on the time to exhaustion within both conditions when compared to the no fluid ingestion trial as presented in Figure 3. The exercise capacity during the graded exercise test within both NF and FI trials was significantly shorter in WH than WD (9.1 ± 2.8 min vs. 12.7 ± 2.4 min, respectively; *p* = 0.01). At exhaustion, T_re_ was not significantly different between the FI and the NF or WD and WH trials (global mean 39.3 ± 0.5°C; all *p* > 0.2). Similarly, T_sk_ (global mean 32.1 ± 1.2°C; all *p* > 0.1) and heart rate (global mean 182 ± 1 beats min^-1^; all *p* > 0.3) were also not significantly different between any condition.

## Discussion

To our knowledge, this is the first study to directly examine the efficacy of fluid ingestion in attenuating thermoregulatory strain during prolonged exercise in two different environments: warm-dry (WD) and warm-humid (WH). This study was designed to simulate real outdoor running where runners would experience a high level of air velocity during their intense running exercise, thus promoting convective cooling. The main finding of this study was that fluid ingestion during exercise in warm-humid and warm-dry conditions did not attenuate hyperthermia during exercise. Consequently, the time to exhaustion in the subsequent graded exercise session was not improved during the FI trial. Under this current scenario, fluid ingestion was seen not to provide any beneficial effect on thermoregulatory and circulatory responses and consequently on exercise performance.

This study had also postulated that the level of percent body mass deficit did not influence the magnitude of physiological strain during submaximal exercise and running performance during the graded exercise test. In the present experiment, the percent body mass deficit during the no fluid (NF) trial, in the WD and WH condition was 2 and 1.8%, respectively.

Therefore, the current study provides evidence that thermoregulatory responses during the submaximal exercise could have been influenced by the level of RH as previously reported (Maughan et al., 2012; Moyen et al., 2014; Che Muhamed et al., 2016) and not associated with the level of dehydration as advocated in numerous publications (Noakes, 2007; Goulet, 2011, 2013; Wall et al., 2013). This current observation is contrary to earlier observations conducted in drier heat that fluid ingestion attenuates hyperthermia during exercise in a heat stressful condition (Montain and Coyle, 1992; Below et al., 1995; Armstrong et al., 1997; Sawka et al., 2012).

The findings of the present study provide new insight in better understanding the efficacy of fluid ingestion on thermoregulatory responses in two different heat stress conditions (i.e., warm-dry and warm-humid). Numerous earlier guidelines on fluid ingestion (Casa et al., 2000; Sawka et al., 2007, 2015) do not specifically comment on the efficacy of fluid ingestion in attenuating heat strain under different thermal profiles. The present study demonstrated that in the humid condition with a reduced E_max_, there is an increase in the amount of sweat dripping off the skin instead of being evaporated (Candas et al., 1979; Frye and Kamon, 1983; Alber-Wallerström and Holmér, 1985). Therefore, the notion that fluid ingestion during exercise heat stress will attenuate hyperthermia by enhancing sweat evaporation does not take place in a humid condition. Instead, the physical characteristics of the environment would dictate the efficacy of fluid ingestion in attenuating hyperthermia. This was evident in the current study where a higher WBSR recorded in the humid condition did not result in a lowering of skin temperature that would have increased the gradient between the core and the skin, thus promoting heat transfer and lowering body heat storage.

Instead, skin temperature remained elevated in the humid condition due to the lower E_max_. The current observation that fluid ingestion was not effective in attenuating thermal strain and improving endurance performance was consistent with several earlier studies. Kay and Marino (2003) reported that water ingestion failed to improve self-paced exercise performance in a hot-humid environment. In addition, several other field-based studies conducted in hot-humid environments had reported no association between fluid intake and percent dehydration with core temperature response (Byrne et al., 2006; Lee et al., 2010). This further supports numerous reports that have proposed that a certain amount of dehydration is tolerable during running exercise where thermoregulatory function is not significantly impaired until a critical level of dehydration has accrued. Davies and Thompson (1986) recorded a 5.5% body weight deficit at the end of 4 h strenuous running exercise with core temperature at no point exceeding 39.3°C. Based on this evidence it is suggested that the level of dehydration may have a relatively small influence on core temperature during exercise if the subjects are euhydrated at the onset of exercise. Several more recent findings have consistently demonstrated that a body mass loss of up to 4% has been well tolerated during prolonged exercise and did not appear to have any detrimental effect on exercise performance (Zouhal et al., 2011; Del Coso et al., 2014; Hoffman and Stuempfle, 2014).

Guidelines on fluid ingestion during exercise have been formulated mainly based on the notion that dehydration impairs endurance exercise performance (Casa et al., 2000; Sawka et al., 2007, 2015) as dehydration level exceeds 2% of body mass (Montain and Coyle, 1992; Below et al., 1995; Ebert et al., 2007; Merry et al., 2010; Sawka et al., 2012; Cheuvront and Kenefick, 2014). On the contrary, several other researchers have consistently shown that in well-trained cyclists who were euhydrated at the onset of exercise and went on to cycle for up to 60 min, their cycling performance was not compromised within an ambient temperature of up to 33°C and 60% RH despite their dehydration level reaching 4% (Noakes, 2007; Goulet, 2011, 2013; Wall et al., 2013).

The level of plasma volume in this study was well maintained during exercise despite the difference in dehydration rate between the NF and FI trial. This observation is consistent with several earlier studies that have shown plasma volume can be partially defended at an even larger percentage of body weight deficit during intense (65–75% 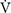
O_2_ max) running exercise (Costill et al., 1970; Sawka et al., 1980; Kolka et al., 1982; Gass et al., 1983; Davies and Thompson, 1986) The magnitude of dehydration in the present study was much smaller than these earlier studies that have reported a stable plasma volume. For instance, Costill et al. (1970), Sawka et al. (1980), and Kolka et al. (1982) recorded a stable plasma volume during exercise at an even higher percent body weight deficit of 4%, 4.8–6.8% and 7%, respectively. The ability to defend the level of plasma volume in light of increasing dehydration level has been associated with the participants euhydration level at the onset of exercise as well as the ability to shift body water interstitially. In addition, the release of water from glycogenolysis, metabolic water production and the redistribution of water from inactive skeletal muscle has also been reported to assist in maintaining plasma volume during exercise (Pivarnik et al., 1984).

## Conclusion

In conclusion, the current study has demonstrated that the efficacy of fluid ingestion in attenuating thermoregulatory and circulatory stress during prolonged exercise is potentially dependent on the physical characteristics of the environment. Runners who started exercise in a euhydrated state did not gain any ergogenic benefit for the 1 h of exercise from fluid ingestion. Runners in our study who had experienced dehydration ranging from 1.8% to 2.0% did not improve their performance. This was in contrast with the well-established notion that dehydration of up to 2% of body weight loss will increase thermoregulatory and circulatory stress leading to impairment in endurance exercise performance (Casa et al., 2000; Sawka et al., 2007, 2015). Instead, our finding was in agreement with earlier reports of Kay and Marino (2003) as well as Lee et al. (2010) which failed to observe any ergogenic benefit of fluid ingestion on exercise performance in humid heat.

This study highlights the need to consider the characteristics of the environment in evaluating the efficacy of fluid ingestion in attenuating thermoregulatory and circulatory strain during exercise and its implications on exercise performance. Future recommendations relating to fluid ingestion during exercise should now discuss the impact of environmental conditions.

## Ethics Statement

The University of Sydney Human Ethics Committee approved the experimental protocol for this study (Ref. No: 99/05/46), which conformed to the current Declaration of Helsinki guidelines.

## Author Contributions

ACM, SS, and MT involved in the design and implementation of the study. TM and HY assisted the corresponding author in the data analysis. All authors contributed in preparing the manuscript with HY preparing the figures.

## Conflict of Interest Statement

The authors declare that the research was conducted in the absence of any commercial or financial relationships that could be construed as a potential conflict of interest. The reviewer ZS declared a past co-authorship with one of the authors TM to the handling Editor.
